# Dapagliflozin promotes white adipose tissue browning though regulating angiogenesis in high fat induced obese mice

**DOI:** 10.1186/s40360-024-00747-5

**Published:** 2024-03-19

**Authors:** Lin Xiang, Min Liu, Guangda Xiang, Ling Yue, Junxia Zhang, Xiaoli Xu, Jing Dong

**Affiliations:** 1 Department of Endocrinology, General Hospital of Central Theater Command, Wuluo Road 627, Wuhan, Hubei Province 430070 China; 2 Department of Endocrine and Metabolic Diseases, Shanghai Institute of Endocrine and Metabolic Diseases, Ruijin Hospital, Shanghai Jiao Tong University School of Medicine, 197 Rujin 2nd Road, Shanghai, China

**Keywords:** Dapagliflozin, Obesity, Adipocytes, White adipose tissue browning

## Abstract

Browning of white adipose tissue (WAT) is become an appealing target for therapeutics in the treatment of obesity and related metabolic diseases. Dapagliflozin is widely used in the treatment of type 2 diabetes, and it is also found that the drug exhibits regulate systemic metabolism such as obesity, insulin resistance and hepatic steatosis. However, the precise role of dapagliflozin on WAT remodeling remains to be elucidated. The current study aimed to explore the role of dapagliflozin on WAT browning in high-fat diet (HFD)-induced obese mice. Male C57BL/6J mice (*n* = 6 per group) were used to establish obesity model by following feeding with HFD for 6 weeks. The mice were randomly treated with or without dapagliflozin for the experimental observation. The volume and fat fraction of WAT were quantified, H&E, UCP-1 staining and immunohistochemistry were conducted to investigate the white-to-brown fat conversion and angiogenesis in WAT respectively. Quantitative real-time polymerase chain reaction (qPCR) was employed to explore the mRNA expression levels of genes related to fat browning and angiogenesis in WAT. Subsequently, 3T3-L1 cells were used to explore the effect of dapagliflozin on preadipocytes differentiation in vitro. Our results demonstrated that dapagliflozin could reduce body weight gain and promote WAT browning in HFD induced obese mice via regulating lipogenesis and angiogenesis in WAT. Furthermore, dapagliflozin reduce cells differentiation, up-regulate the expression of WAT browning and angiogenesis genes in 3T3-L1 adipocytes in vitro. In conclusion, dapagliflozin can potentially promote WAT browning in HFD induced obese mice via improving lipogenesis and angiogenesis in WAT.

**Supplementary Information:**

The online version contains supplementary material available at 10.1186/s40360-024-00747-5.

## Introduction

Obesity is the pathophysiological condition characterized as excessive or abnormal accumulation of fat, and regard as an increased risk for varies chronic diseases such as hypertension, hypercholesterolemia, diabetes, cardiovascular disease and cancers [1, 2]. Long-term excessive energy storage in white adipose tissue (WAT), including visceral adipose tissue and subcutaneous adipose tissue, is considered a major cause of obesity [3]. On the contrary, brown adipose tissue (BAT) serves as a thermogenic tissue and expends energy by consuming glucose and fatty acids. The strategies to increase energy expenditure, stimulating the development of beige adipocytes in WAT (so called ‘browning’) represent an attractive concept for combating obesity and associated metabolic diseases [4].

Dapagliflozin exert the glucose-lowering effect through inhibition of the Sodium-glucose cotransporter (SGLT) 2 protein in the kidney proximal tubule, resulting in the excretion of glucose and calories into the urine [5]. Multiple pre-clinical and clinical studies have demonstrated that SGLT2 inhibitors are benefit for patients with cardiovascular disease, coronary artery disease, and heart failure with or without reduced ejection fraction [6, 7]. Recent clinical studies also indicated that SGLT2 inhibitors associated with improvement in hepatic fat content, a decrease in visceral fat and body weight among diabetic patients [8, 9]. Furthermore, it is reported that SGLT2 inhibitors improves lipid profiles and reduces lipid accumulation in mice [7]. Yet the mechanisms underlying these effects are not fully elucidated.

We hypothesized that dapagliflozin treatment improve obesity through promotes white adipose tissue browning in high fat diet (HFD) induced obese mice. Thus, we investigated the effect of dapagliflozin on HFD induced obese mice and explored its possible mechanisms.

## Materials and methods

### Animal model

Male C57BL/6J background (age, 3–4 weeks) mice were purchased from Shanghai Model Organisms Centre, Inc. Mice with similar body weights (20 ± 2 g) were allowed to adjust to the environment for the experiments. Then the mice (age, 4–5 weeks) were randomly grouped into three groups (*n* = 6 per group): the normal chow diet (ND) group; the HFD group; and the HFD + dapagliflozin (Dapa) group. Mice in HFD group were fed with a diet containing 60% calories as fat (D12492, 60% fat, 20% carbohydrate and 20% protein; Research Diets, New Brunswick, NJ). Mice in ND group were fed with the standard chow diet (CD; D12450B, 10% fat, 70% carbohydrates, and 20% protein, Research Diet, New Brunswick, NJ). Following feeding for 6 weeks, mice with HFD were randomly allocated to the test groups for pharmacological studies. Mice in the dapagliflozin group were intragastrically administered with 1 mg/kg/day dapagliflozin for 9 weeks according to preliminary experiments and previous studies [10, 11]. Mice in the other groups were treated with the same volume of 0.5% sodium carboxyl methyl cellulose solution. At the end of the experiment, all mice were euthanatized by cervical dislocation. All mice were housed in specific pathogen-free (SPF) facility under a 12-h light/dark cycle, a relative humidity of 50% and a controlled temperature of 22 ± 1 °C. All animal procedures were approved by the Animal Care and Use Committee of the General Hospital of Central Theater Command.

### Serum biochemical measurements

Blood glucose levels were detected using a blood glucose meter (Bayer). The serum levels of triglycerides (TG) were measured using an automatic biochemical analyzer (Hitachi, Ltd.). In addition, the serum levels of free fatty acids (FFA) and insulin were detected by a commercially available colorimetric assay kit (Nanjing Jian Cheng Bioengineering Institute).

### Histology and immunofluorescence

Inguinal adipose tissues (iWAT) and epididymal adipose tissues (eWAT) from mice in each group were harvested and fixed in 4% paraformaldehyde. After 24 h, the tissues were washed with phosphate buffered saline (PBS), followed by dehydration and embedded in paraffin. Subsequently, sections of the embedded tissues were subjected to H&E and UCP-1 staining. For immunofluorescence staining, sections of the embedded tissues were incubated with anti-rabbit CD31 antibody (1:200, ABclonal, A2104) overnight at 4 ◦C, followed by staining with an Alexa Fluor 647-conjugated goat anti-rabbit IgG (1:400, Abcam, #ab150079) for 1 h at 4℃. Then, the sections were stained with 10 µg/mL Hoechst for 10 min and washed three times with PBS. Finally, the cell culture slides were mounted onto the slides and examined under a confocal laser scanning microscope (Carl Zeiss, Jena, Germany).

### Cell culture

Mouse 3T3-L1 cells were proliferated in Dulbecco’s modified Eagle’s medium (DMEM) supplemented with 10% fetal bovine serum. Cells were grown to confluency for inducing cell differentiation into adipocytes. Briefly, cells were incubated in a differentiation medium containing 0.5mM 1-methyl-3-isobutyl-xanthine, 1µM dexamethasone, and 1 µg/ml insulin (MDI) in DMEM with 10% fetal bovine serum for 2 days. Thereafter, cells were maintained in DMEM with 10% FBS for absolute differentiation. Cells were treated with palmitic acid (0.6 mM, Sigma-Aldrich) for 24 h to induce lipid accumulation. The medium was changed every 2 days. Cells were stained on day 8 with H&E and Oil Red O staining.

### Cell viability

Ninety microliters (5 × 10^4^/mL) of 3T3-L1 adipocytes were seeded into 96-well plates. After 24 h, when the adipocytes reached ∼80% confluency, the cells were treated with various concentrations of dapagliflozin for an additional 24 h. Cell viability was assessed using a Cell Counting Kit 8 assay kit (Wuxi Crondabio Technology).

### Western blot analysis

**Fig. 1:**
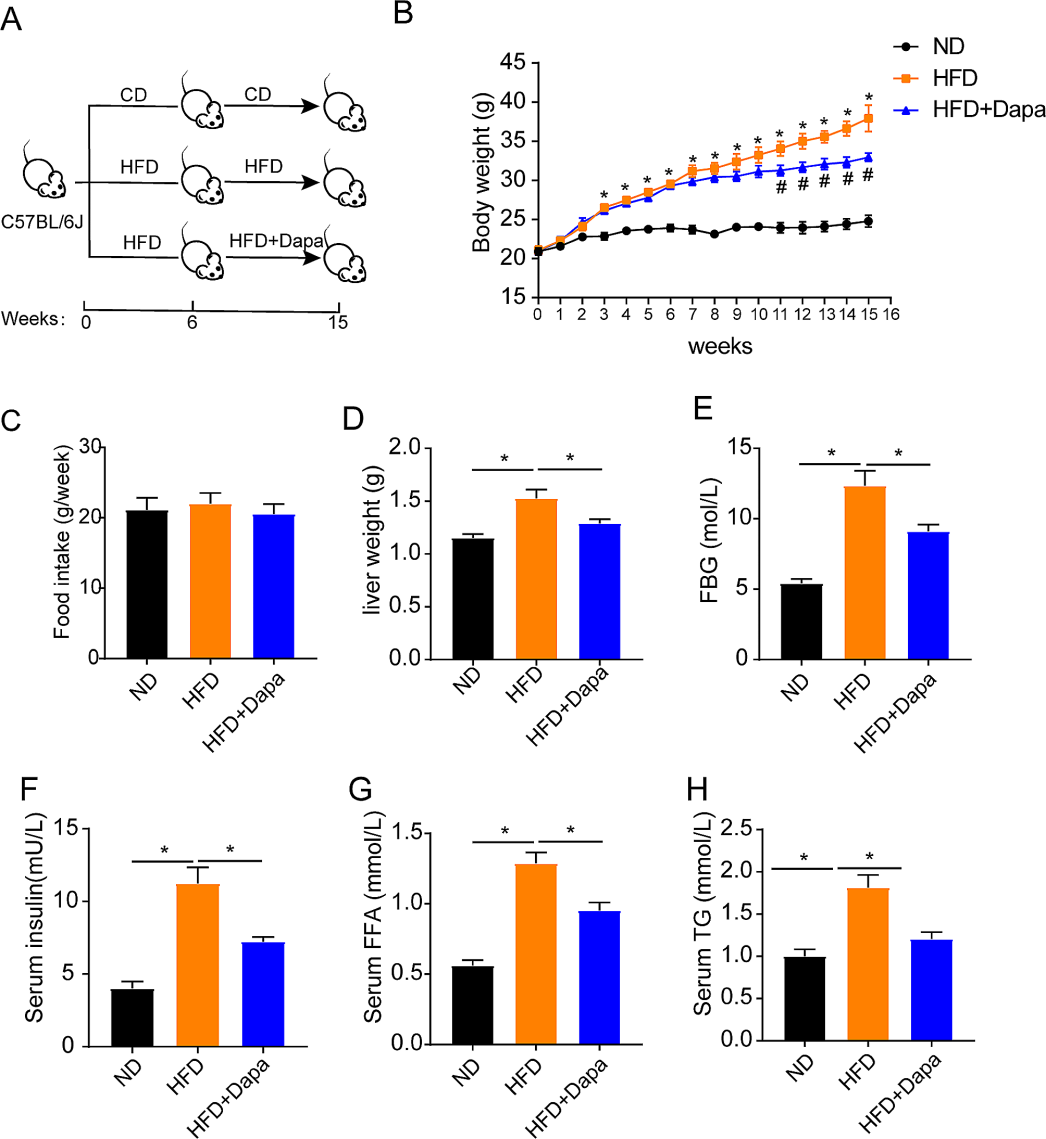
Effects of dapagliflozin on body weight and glycolipid metabolism in mice. (**A**) Schematic study plan. 4–5 weeks old, male C57BL/6 mice with similar body weights were fed with normal chow diet (CD) or high fat diet (HFD). After 6weeks, mice in HFD + Dapa group were intragastrically administered with 1 mg /kg /day dapagliflozin (Dapa) for 9 weeks, mice in other groups were treated with 0.5% sodium carboxyl methyl cellulose solution as controls. (**B**) Body weight change. (**C**) The effect of 9 weeks of intragastric administration of Dapa on food intake. (**D**) Liver weight. (**E**) Levels of fasting blood glucose (FBG). (**F**–**H**) Levels of insulin, FFA and TG in serum. (*n* = 5). Values are mean ± SEM. *, *P* < 0.05

Proteins were extracted from cells and abdominal adipose tissue using ice-cold RIPA buffer. Total proteins were separated by SDS–PAGE and then transferred onto PVDF membranes. The protein bands were incubated with primary antibodies against the following proteins: UCP-1 (ab234430, 1:1000), PGC-1α(ab176328, 1:1000) at 4℃, The next day, the blotted were incubated with a specific secondary antibody (1:2000, Beyotime, Shanghai, China). GAPDH was used as an internal marker. The primers sequences used are listed in S-Fig. 1. The intensity of protein bands was performed using the Image J software.

### Quantitative real-time PCR analysis

Total RNA was extracted from adipose tissues or cells using the Trizol reagent, and was then reverse transcribed into cDNA. qPCR was performed using the SYBR Green PCR master mix (Applied Biosystems; Thermo Fisher Scientific, Inc.). The primers sequences used are listed in S-Table 1. The mRNA expression levels of target genes were calculated using the 2-ΔΔCq method and normalized to those of GAPDH.

### Statistics analysis

All the data were expressed as the mean ± standard error of the mean (SEM). Comparisons between two groups were performed using Student’s t-test. *P* < 0.05 was considered significant.

## Results

### Effects of dapagliflozin on body weight and glycolipid metabolism in mice

To confirm the influence of dapagliflozin on body weight gain and the metabolic phenotypes in mice. Male C57BL/6 mice with similar body weights were fed with ND or HFD and were given 1 mg/kg dapagliflozin (Dapa) or equivalent amount of solvent as control from the 6th week (Fig. 1A**)**. Body weights were weekly assessed in different groups. As shown in Fig. 1B, the body weights of mice in the HFD + Dapa group was notably reduced compared with that in the HFD group, however the food intake between different groups were not significantly altered (Fig. 1C**)**. Furthermore, HFD-treated mice exhibited elevated liver weights and fasting blood glucose when compared with ND control mice. However, the high weights of liver and high level of glucose in HFD mice were abrogated by dapagliflozin treatment (Fig. 1D-E). In addition, the serum levels of insulin, FFA and TG were significantly decreased in HFD mice treated with dapagliflozin (Fig. 1F-H). These findings indicated that dapagliflozin could reduce body weight gain and improve the glycolipid metabolism in HFD mice.

### Dapagliflozin improves lipogenesis and induces white adipose tissue (WAT) browning in mice

**Fig. 2:**
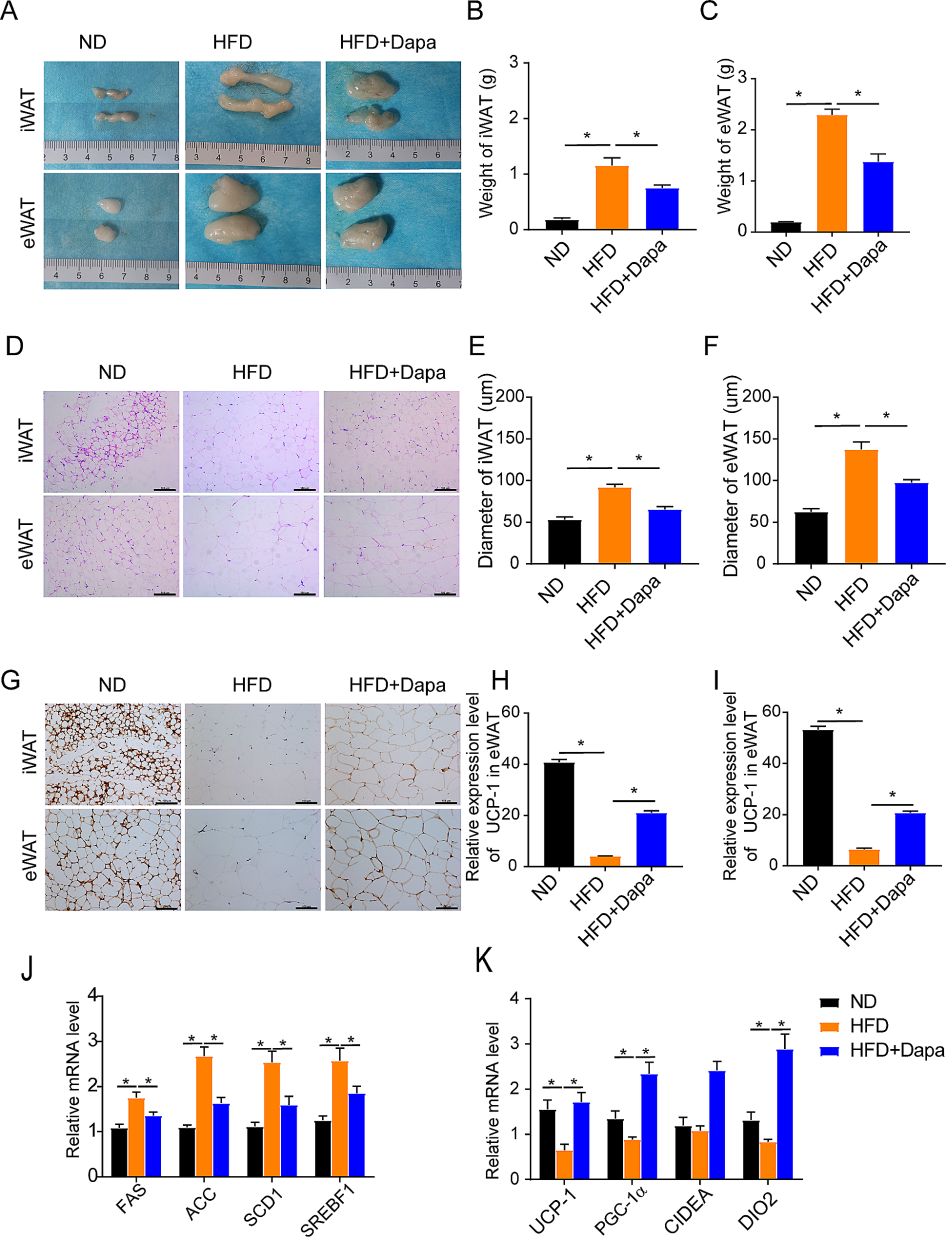
Dapagliflozin improves lipogenesis and induces white adipose tissue (WAT) browning in mice. (**A-C**) Representative images and weight of iWAT and eWAT in different groups. (**D-F**) H&E staining of iWAT and eWAT (Scar bar 100 μm) and the size of adipocytes in different groups. (**G-I**) Immunohistochemical staining of iWAT and eWAT (Scar bar 100 μm) and relative expression of UCP-1 in different groups. (**J**) Relative mRNA expression of lipogenesis -related genes in WAT. (**K**) Relative mRNA expression levels of brown gene in WAT. (*n* = 5). Values are mean ± SEM. *, *P* < 0.05

Subsequently, the present study investigated whether dapagliflozin had an impact on adiposity phenotypes in HFD mice. Results showed that dapagliflozin administration could slightly attenuated subcutaneous (iWAT) and epididymal WAT (eWAT) mass as compared to the HFD mice treated with control (Fig. 2A-C**)**.

We next analyzed the morphology of WAT and the expression of thermogenic protein UCP-1 in WAT. Histological examination showed that the diameter of fat cells in HFD mice increased and the number of cells per unit area decreased compared with the ND mice. However, the adipocytes of the mice treated with dapagliflozin were smaller and more tightly packed compared to the HFD mice (Fig. 2D-F**)**. The expression level of UCP-1protein were also increased with dapagliflozin treatment (Fig. 2G-I**)**. Furthermore, the mRNA abundance of lipogenesis -related genes (FAS, ACC, SCD1, SREBF1) were markedly down-regulated by treatment with dapagliflozin of HFD mice (Fig. 2J**)**. Importantly, the mRNA expression levels of genes involved in fat browning in iWAT including UCP-1, PGC1a, CIDEA, and DIO2 were significantly up-regulated after dapagliflozin administration (Fig. 2K**)**. Taken together, the aforementioned results suggested that dapagliflozin could improves lipogenesis and induces WAT browning in HFD mice.

### Dapagliflozin regulates adipose tissue angiogenesis in WAT

**Fig. 3:**
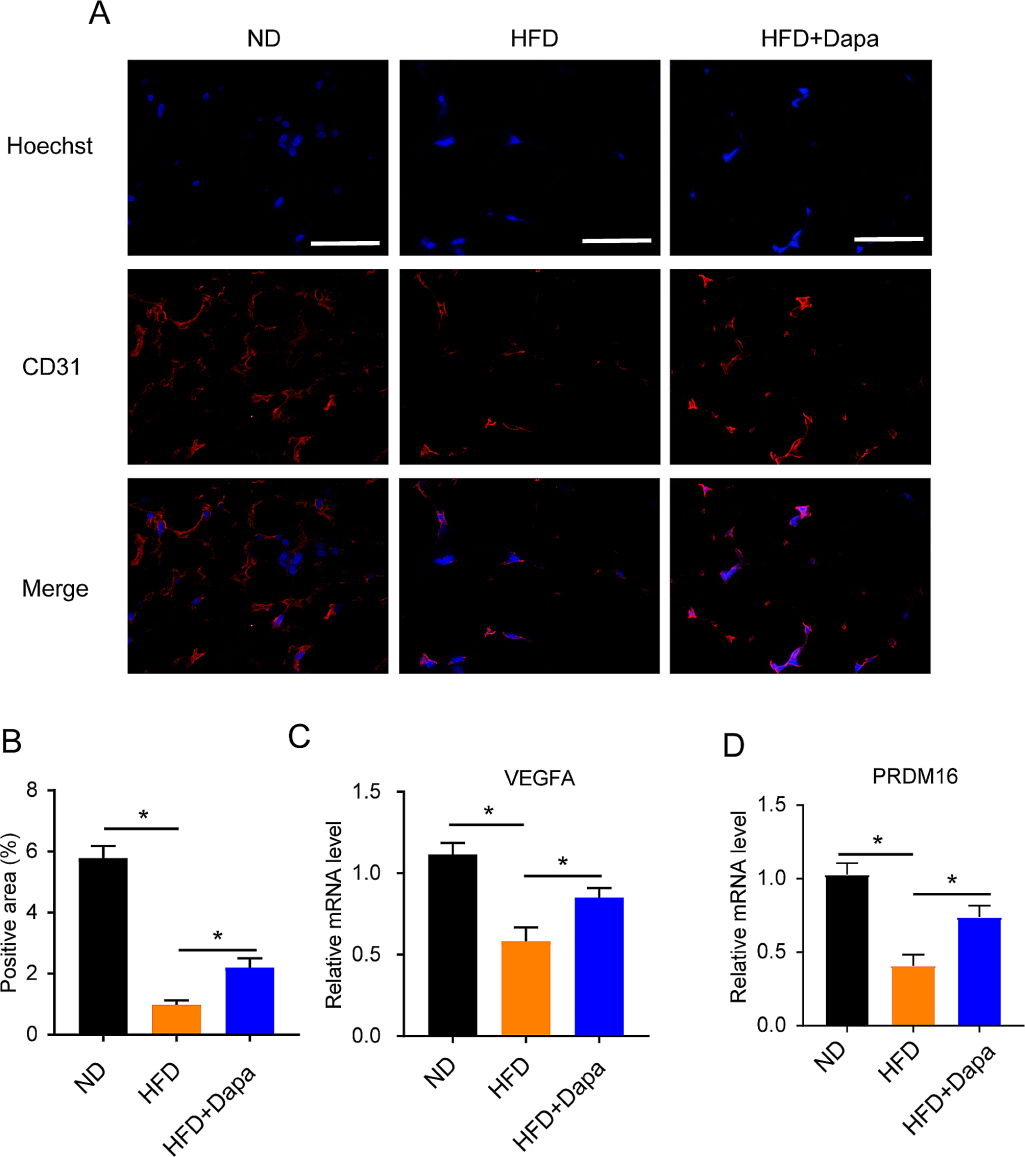
Dapagliflozin regulates adipose tissue angiogenesis in WAT. (**A-B**) Representative immunofluorescence images and relative and relative fluorescence intensive level of CD31 in WAT from different group mice. Scar bar 50 μm. (**C-D**) Relative mRNA expression of VEGFA and PRDM16 in WAT from different group mice. (*n* = 5). Values are mean ± SEM. *, *P* < 0.05

To further determine whether dapagliflozin affect adipose tissue angiogenesis in WAT, here, WAT tissue sections were stained for CD31, an endothelial cell marker. Results showed a significant reduction in capillary density (angiogenesis) as CD31 expression in WAT from HFD mice, notably, these changes were reversed in dapagliflozin treatment mice (Fig. 3**A-B)**. In addition, the mRNA expression levels of VEGFA and PRDM16 were also increased (Fig. 3**C-D)**. These data demonstrated that dapagliflozin promotes white adipose tissue browning involved with up-regulated adipose tissue angiogenesis.

### Dapagliflozin reduced the differentiation of 3T3-L1 preadipocytes

**Fig. 4:**
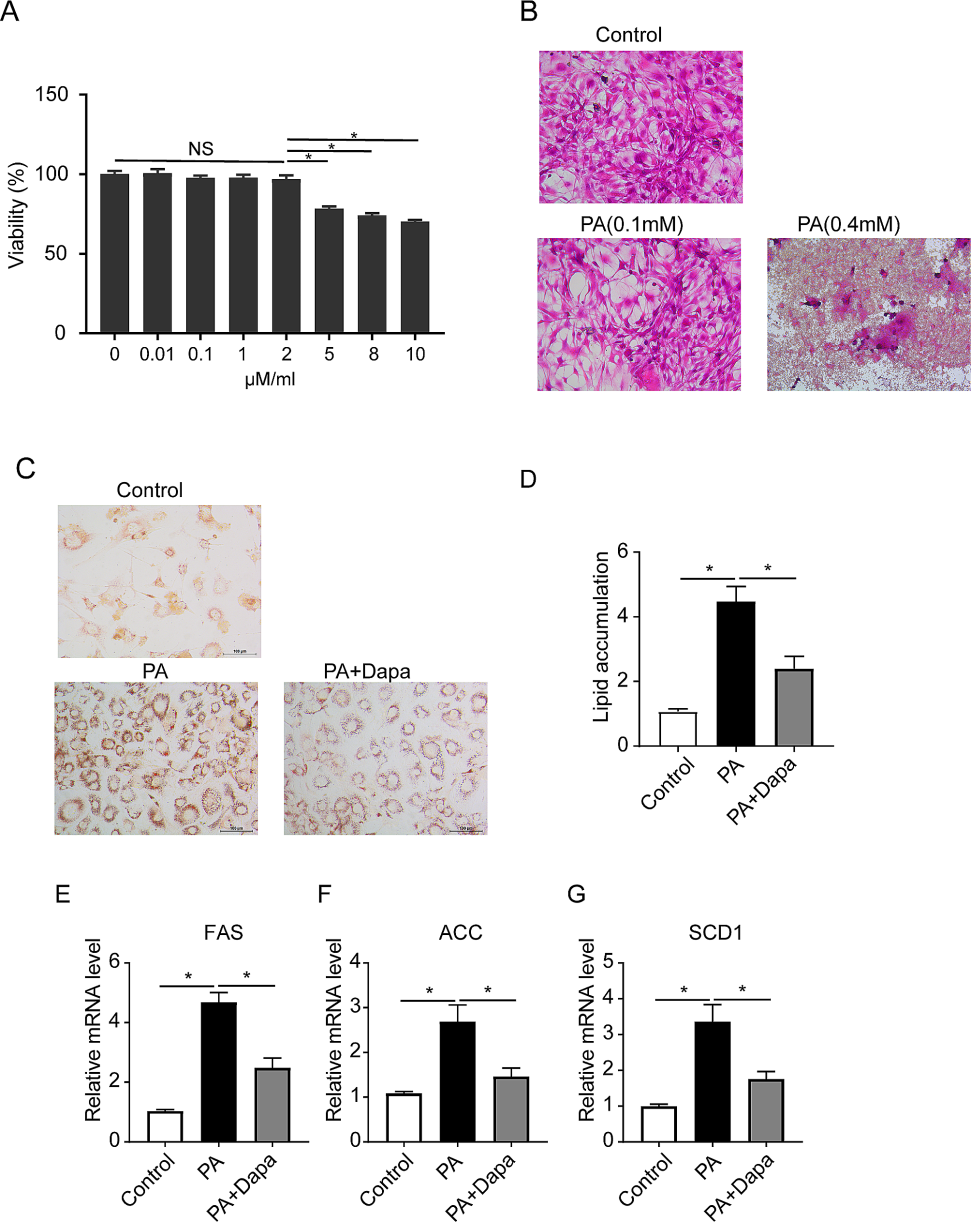
Dapagliflozin reduced the differentiation of 3T3-L1 preadipocytes. (**A**) The effect of dapagliflozin on cell viability. (**B**) H&E staining of adipocytes under different concentration PA intervention. (**C**) Oil Red O staining of differentiated adipocytes. (**D**) Relative lipid accumulation in different group cells as examined by measuring absorbance at 540 nm of Oil Red O staining. (**E-G**) Relative mRNA expression of lipogenesis -related genes in cells. Values are mean ± SEM. *P < 0.05. Each experiment in vitro was repeated 3 times

We next aimed to explore whether dapagliflozin could exert an effect on 3T3-L1 preadipocytes. Firstly, CCK8 assay was used to detect the toxic effect of dapagliflozin on 3T3-L1 preadipocytes. The results showed that the cell viability of 3T3-L1 cells was diminished when dapagliflozin concentration was above 2 µM/ml (Fig. 4A**)**. Secondly, cells were treated with palmitic acid (PA) to induce lipid accumulation. We also observed the effect of different concentrations of PA on cell growth, and the results showed that 0.1mM/L PA was the optimum condition (Fig. 4B**)**. Therefore, we choose 2 µM/ml dapagliflozin and 0.1mM/L PA as the optimum concentration for the following experiment. As shown in Fig. 4C-D , the lipid accumulation was significantly elevated by stimulation with PA, but was dramatically suppressed by the introduction of dapagliflozin. Of note, the expression levels of lipogenesis -related genes including FAS, ACC and SCD1 were significantly enhanced in 3T3-L1 cells under PA treatment, while dapagliflozin treatment reversed these effects in the cells (Fig. 4E-G**)**. The findings indicated that dapagliflozin reduced the differentiation and lipogenesis in 3T3-L1 preadipocytes.

### Dapagliflozin regulated genes expression of fat browning and angiogenesis in adipocytes in vitro

**Fig. 5:**
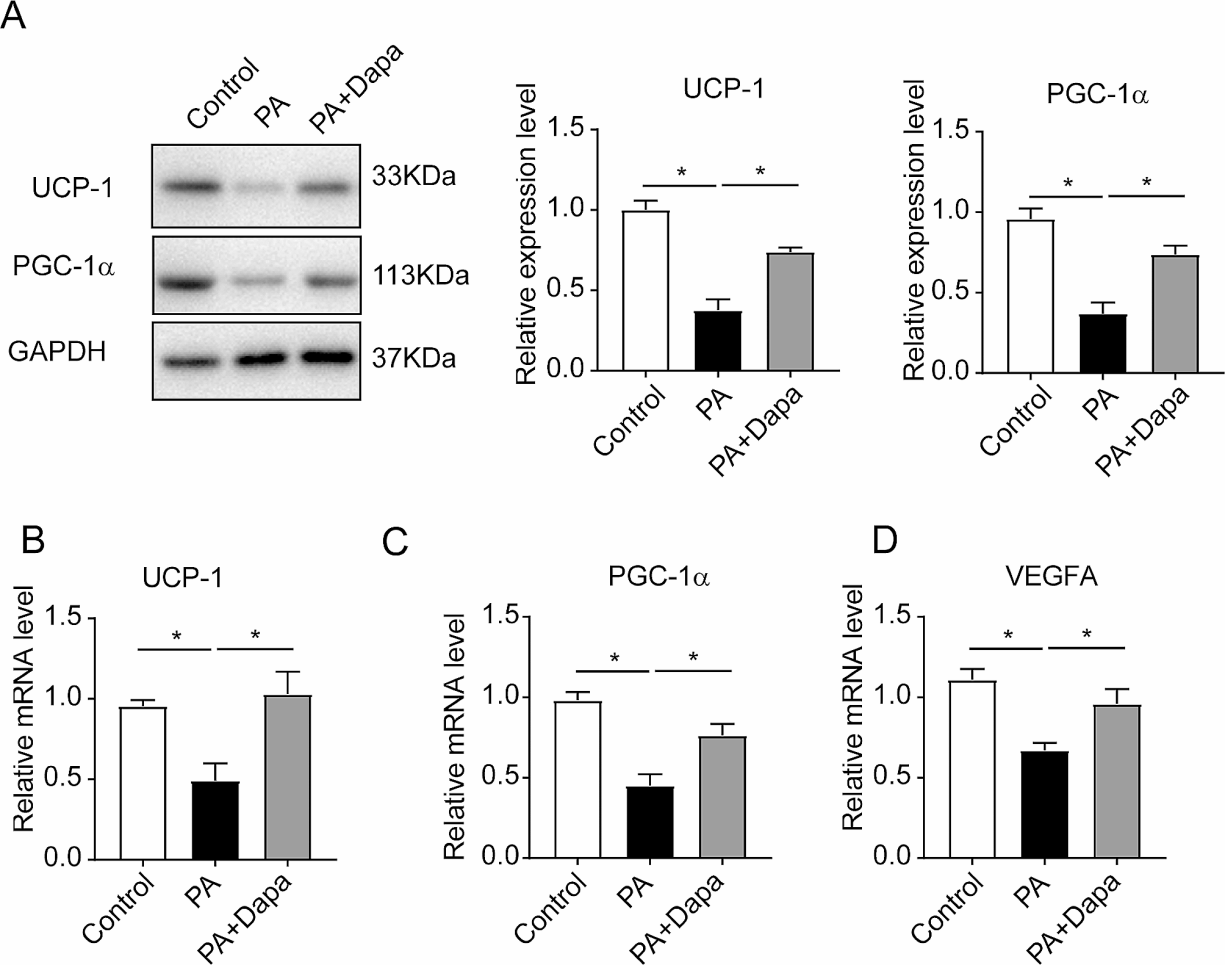
Dapagliflozin regulated genes expression of WAT browning and angiogenesis in adipocytes in vitro. (**A**) Representative WB images and the protein levels of UCP-1 and PCG-1α. (**B-C**) Relative mRNA expression levels of brown gene in 3T3-L1 cells (**D**) Relative mRNA expression levels of VEGFA in 3T3-L1 cells. Values are mean ± SEM. *P < 0.05. Each experiment in vitro was repeated 3 times

Finally, we also verified the function of dapagliflozin on browning and angiogenesis in 3T3-L1 cells. Here, results showed that the UCP-1 and PCG-1α protein expression were suppressed by PA compared to vehicle; however, dapagliflozin intervention can reverse these effects (Fig. 5A**)**. Consistent with the results in vivo, dapagliflozin intervention increased the mRNA expression levels of browning genes such as UCP-1and PCG-1α (Fig. 5B-C**)** and angiogenesis gene VEGFA (Fig. 5D**)**. These results suggested that dapagliflozin regulated genes expression of fat browning and angiogenesis in vitro.

## Discussion

Several studies have demonstrated that accumulation of excess WAT has deleterious consequences for metabolic health, but the activation of BAT confers beneficial effects on adiposity, insulin resistance and hyperlipidaemia [12, 13]. Regulating WAT differentiation to promote brown fat production is a new approach to obesity treatment. Our study aimed to investigate the effect of dapagliflozin on adipose tissue and the underlying molecular mechanism by which dapagliflozin induces WAT browning in HFD induced obese mice. The main findings of the present study were as follows: (i) dapagliflozin could reduce body weight gain and improve the glycolipid metabolism in HFD mice. (ii) dapagliflozin could play a beneficial role in obesity via regulating lipogenesis and angiogenesis. (iii) dapagliflozin could reduce cells differentiation, up-regulate expression of fat browning and angiogenesis genes in 3T3-L1 adipocytes in vitro.

Dapagliflozin is the most selective and orally active SGLT2 inhibitor for clinical application, and demonstrated a significant reduction in kidney and cardiovascular end points with dapagliflozin use [14, 15]. Previous studies have provided several evidence on the promising impacts of SGLT2 inhibitors on metabolic disorders including obesity, insulin resistance and diabetes mellitus [16, 17]. Recently, the regulatory effect of dapagliflozin on WAT is also gaining increasing attention by researchers. Clinical studies have shown that treatment with dapagliflozin might improve systemic metabolic parameters and decrease the epicardial adipose tissue (EAT) volume in diabetes mellitus patients [18, 19]. Eva Kralova et al. found that dapagliflozin influenced visceral fat gene expression in streptozotocin-induced diabetes mellitus Wistar rats. Furthermore, Tuo Han et al. [20]. reported that dapagliflozin induced gonadal adipose tissue (gWAT) browning and improved local oxidative stress, thus inhibiting fat accumulation and hepatic steatosis in obese mice. Consitently, the results of our study further confirmed that dapagliflozin induced WAT browning in HFD induced obese mice and we then explored the possible mechanisms. At present, it is identified that the increased expression levels of adipogenic-specific genes including SREBF1, FAS, ACC and SCD1 are associated with HFD-induced obesity [21, 22]. Moreover, the possible influencing factors of browning of white fat have been found, genes such as UCP-1, PGC‑1α, CIDEA and DIO2 are considered to be important regulatory factors, which can improve mitochondrial function, enhance heat production capacity to promote the browning of white fat [23, 24]. Our finding further demonstrated that dapagliflozin regulating lipogenesis and browning genes of WAT in vivo and in vitro.

In any tissue, proper vascularization and blood perfusion are compulsory for its growth, expansion, and metabolic status [25]. Angiogenesis also plays a significant role in adipogenesis, as it significantly decreases once white adipose tissue maturation is complete, leaving behind a hypoxic microenvironment during adipogenesis. Importantly, recent studies also shown that cold-induced sympathetic activation markedly augments adipose angiogenesis during browning of subcutaneous WAT and VEGF is the key angiogenic mediator in this experimental setting [26, 27]. Our data indicated that dapagliflozin improved adipose tissue angiogenesis and up-regulated mRNA expression levels of VEGFA. Previous studies have also explored the signaling pathway mechanism governing angiogenesis in adipose tissue browning. Some studies showed that Apigenin can mediate white to brown adipocyte transition via VEGF-PRDM16 signaling [25] and Retinoic acid can activate p38MAPK to enhance the PRDM16 promoter transcription and promote WAT browning. Thus, we further examined the mRNA expression level of PRDM16 gene in adipose tissue of each group. The results showed that dapagliflozin increased the expression of PRDM16 in high fat induced obese mice.

Although the current study shed light on the effect of dapagliflozin promotes WAT browning in HFD induced obese mice, there are still have some limitations. First, the study could not be ruled out that other mechanisms, such as inflammation response, autophagy and ferroptosis could be involved in the process. Second, we did not elucidate the detailed signaling pathway mechanism governing angiogenesis of dapagliflozin mediated adipose tissue browning. Overall, further evidence is needed to clarify the mechanism underlying the effect of dapagliflozin on improving obesity and WAT browning.

In conclusion, the present study demonstrated that dapagliflozin promotes WAT browning in HFD induced obese mice via improving lipogenesis and angiogenesis in adipose tissue. Therefore, the study had successfully proved the promising benefits of dapagliflozin on activate and facilitate browning of WAT, and dapagliflozin is expected for treating obesity-related metabolic diseases and disorders.

### Electronic supplementary material

Below is the link to the electronic supplementary material.

Supplementary Material 1

## Data Availability

The data used and/or analyzed during the current study are available from the corresponding author on reasonable request.
