# Supplementary material for: Dapagliflozin promotes white adipose tissue browning though regulating angiogenesis in high fat induced obese mice
Source: BMC Pharmacol Toxicol. 2024 Mar 19;25:26. doi: 10.1186/s40360-024-00747-5 (PMC10949628; doi:10.1186/s40360-024-00747-5)
Supplement: Supplementary file 1 — Supplementary Material 1 [file 40360_2024_747_MOESM1_ESM.docx]

**S-Table 1. A list of RT-PCR primers used in this study.**

| **Gene** | Forward primer (5’ to 3’) | Reverse primer (5’ to 3’) |
| --- | --- | --- |
| FAS | GGAGGTGGTGATAGCCGGTAT | TGGGTAATCCATAGAGCCCAG |
| ACC | CATGCGATCTATCCGTCGGT | CAGGCACTGGAACATAGTGGT |
| SCD1 | TTCTTGCGATACACTCTGGTGC | CGGGATTGAATGTTCTTGTCGT |
| SREBF1 | GATGTGCGAACTGGACACAG | CAT AGGGGGCGTCAAACAG |
| UCP-1 | GGATTGGCCTCTACGACTCA | GCATTCTGACCTTCACGACC |
| PGC-1α | CAACAGCAAAAGCCACAAAG | ACTGCGGTTGTGTATGGGA |
| GIDEA | CAATGTCAAAGCCACGATGTAC | CTGTGCAGCATAGGACATAAAC |
| DIO2 | CTTCCTCCTAGATGCCTACAAAC | GGCATAAATTGTTACCTGATTCAGG |
| VEGFA | CTGTAACGATGAAGCCCTGGAG | TGGTGAGGTTTGATCCGCAT |
| PRDM16 | CAGCACGGTGAAGCCATTC | GCGTGCATCCGCTTGTG |
| GAPDH | TCAACAGCAACTCCCACTCTTCCA | TTGTCATTGAGAGCAATGCCAGCC |

**S-Figure 1. Full-length gels and blots for our study**


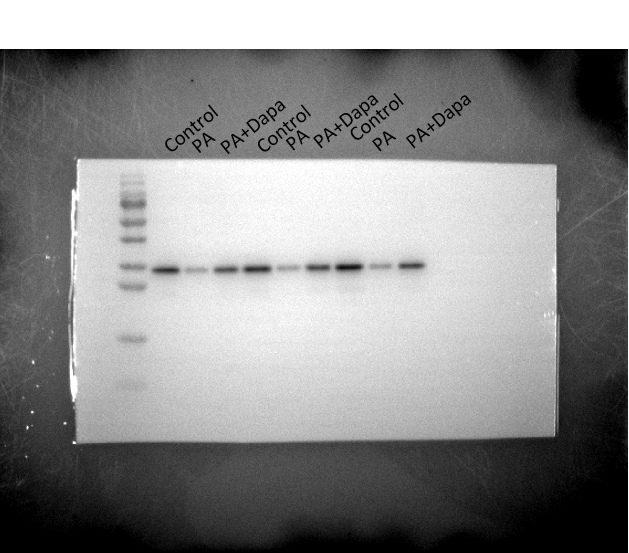


Full-length gels and blots of UCP-1 protein


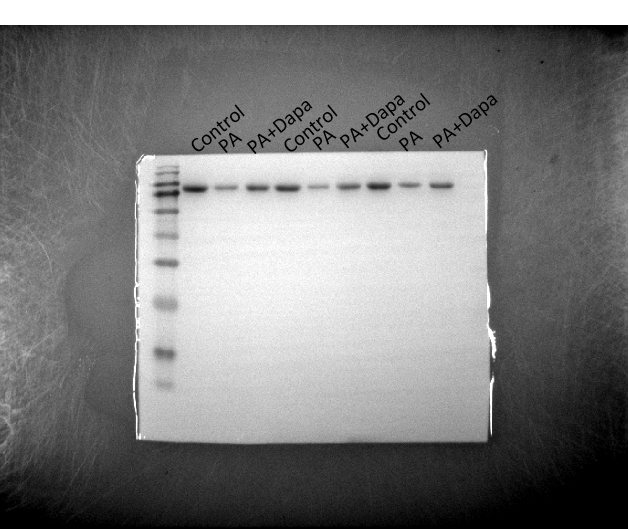


Full-length gels and blots of PCG-1α protein


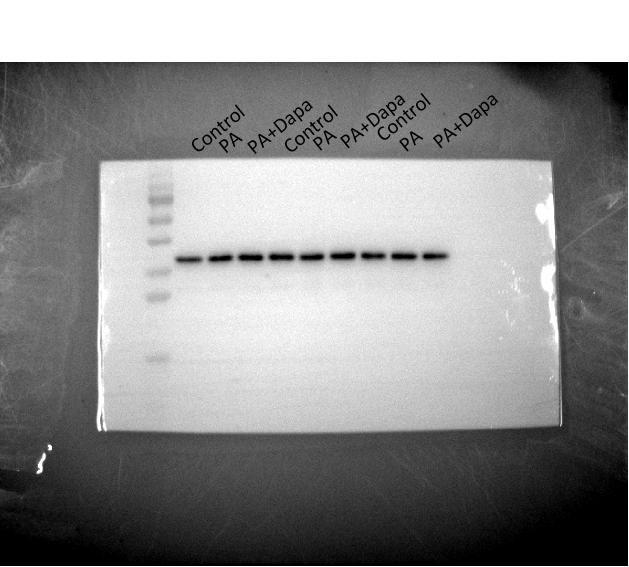


Full-length gels and blots of GAPDH protein
